# 3′-Terminated Overhangs Regulate DNA Double-Strand Break Processing in *Escherichia coli*

**DOI:** 10.1534/g3.117.043521

**Published:** 2017-07-14

**Authors:** Edyta Đermić, Davor Zahradka, Dušica Vujaklija, Siniša Ivanković, Damir Đermić

**Affiliations:** * Department of Plant Pathology, Faculty of Agriculture, University of Zagreb, 10000, Croatia; † Division of Molecular Biology, Ruđer Bošković Institute, 10000 Zagreb, Croatia; ‡ Division of Molecular Medicine, Ruđer Bošković Institute, 10000 Zagreb, Croatia

**Keywords:** DNA degradation, exonuclease activity, RecA protein, single-strand-specific exonucleases, *recB1080* mutant

## Abstract

Double-strand breaks (DSBs) are lethal DNA lesions, which are repaired by homologous recombination in *Escherichia coli*. To study DSB processing *in vivo*, we induced DSBs into the *E. coli* chromosome by γ-irradiation and measured chromosomal degradation. We show that the DNA degradation is regulated by RecA protein concentration and its rate of association with single-stranded DNA (ssDNA). RecA decreased DNA degradation in wild-type, *recB*, and *recD* strains, indicating that it is a general phenomenon in *E. coli*. On the other hand, DNA degradation was greatly reduced and unaffected by RecA in the *recB1080* mutant (which produces long overhangs) and in a strain devoid of four exonucleases that degrade a 3′ tail (ssExos). 3′–5′ ssExos deficiency is epistatic to RecA deficiency concerning DNA degradation, suggesting that bound RecA is shielding the 3′ tail from degradation by 3′–5′ ssExos. Since 3′ tail preservation is common to all these situations, we infer that RecA polymerization constitutes a subset of mechanisms for preserving the integrity of 3′ tails emanating from DSBs, along with 3′ tail’s massive length, or prevention of their degradation by inactivation of 3′–5′ ssExos. Thus, we conclude that 3′ overhangs are crucial in controlling the extent of DSB processing in *E. coli*. This study suggests a regulatory mechanism for DSB processing in *E. coli*, wherein 3′ tails impose a negative feedback loop on DSB processing reactions, specifically on helicase reloading onto dsDNA ends.

A DSB is an adverse DNA lesion that has to be repaired in order for a cell to survive. DSBs are repaired in all living organisms by either mutagenic nonhomologous end joining or by much more universally distributed and precise homologous recombination (HR). During HR, a single 3′-terminated strand is produced from each of two double-strand DNA (dsDNA) ends of a DSB by a process called DNA end resection, wherein a combination of helicase and nuclease activities result in degradation of complementary 5′-terminated strands (Symington 2014). The 3′-end overhangs emanating from a DSB are bound by a recombinase protein, thus creating the central recombination intermediate, the nucleoprotein filament. A recombinase nucleoprotein filament searches for an intact homologous sequence and invades it, hence restoring continuity of genomic information.

Since evolutionarily conserved recombinase proteins [RecA, RadA, and Rad51 (Dmc1) from bacteria, archaea, and eukaryotes, respectively] have a lower affinity of binding to ssDNA than their cognate ssDNA-binding proteins SSB/RPA, a recombination-mediator class of proteins (RecBCD and RecFOR proteins in bacteria and BRCA2, PALB2, and Rad52 in eukaryotes) facilitates recombinase polymerization on ssDNA (Zelensky *et al.* 2014).

In addition to its role in HR, the RecA nucleoprotein filament in *Escherichia coli* serves as a coprotease to promote autocatalytic cleavage of the LexA repressor, leading to induction of an SOS response (Little 1991). RecA also activates a mutagenic DNA polymerase V during SOS induction (Shinagawa *et al.* 1988).

In bacteria, both helicase and nuclease activities for DNA end resection are provided by the functionally related RecBCD, AddAB, and AdnAB enzymes (Wigley 2013). In *E. coli*, the RecBCD enzyme binds to a flush dsDNA end and unwinds a duplex molecule with its fast and processive helicase activity (Dillingham and Kowalczykowski 2008). Both of the unwound strands are degraded by a single nuclease center of the enzyme (residing in its RecB subunit) (Yu *et al.* 1998) until the enzyme encounters a regulatory octanucleotide sequence designated χ. Interaction with χ changes RecBCD’s behavior so that it ceases degradation of the 3′-terminated strand, while continuing DNA unwinding and degradation of the 5′-terminated strand (Anderson and Kowalczykowski 1997a). Also, the χ-modified RecBCD starts facilitating RecA polymerization onto the post-χ 3′ strand, hence producing a RecA-nucleoprotein filament (Anderson and Kowalczykowski 1997b). In this way, χ switches RecBCD enzyme degradation activity into a repair activity.

DSB repair in *E. coli* is active even in the absence of RecBCD due to RecQ helicase unwinding of duplex DNA, RecJ exonuclease trimming of ssDNA tails (ssExo) from the 5′-end, and RecFOR proteins mediating RecA polymerization onto the unwound 3′ overhangs. This pathway is operative only when ssExos that degrade 3′-terminated overhangs [*e.g.*, Exonuclease I (ExoI) and SbcCD] are inactive, which enables stabilization of the recombinogenic substrate (Persky and Lovett 2008).

There are some mutants that show only partial function of RecBCD, yet are DSB repair proficient as well. When RecBCD lacks its RecD subunit, the resulting RecBC enzyme shows reduced helicase rate and processivity compared to RecBCD, and is completely devoid of nuclease activity and χ interaction (Dillingham and Kowalczykowski 2008). In the *recD* mutant, RecBC enzyme unwinds duplex DNA and constitutively loads RecA protein onto the unwound 3′ tail (Churchill *et al.* 1999), while its 5′ complement is trimmed by RecJ and Exonuclease VII (ExoVII) ssExos (Đermić 2006; Đermić *et al.* 2006a).

The *recB1080* mutation renders the RecBCD enzyme deficient in nuclease and RecA loading activity, whereas the enzyme’s binding to DNA as well as rate and processivity of its helicase activity is unaffected (Yu *et al.* 1998; Anderson *et al.* 1999). *In vitro*, the RecB^1080^CD enzyme unwinds the linear DNA duplex, producing full length, RecA-free ss tails in the presence of SSB (Yu *et al.* 1998; Anderson *et al.* 1999). In the *recB1080* mutant, the 5′-ended tail is clipped by RecJ and ExoVII ssExos, while its 3′ complement is covered with RecA protein with the help of RecFOR proteins (Jockovich and Myers 2001; Ivančić-Baće *et al.* 2003; Ivanković *et al.* 2017). The *recB1080* mutant is recombination proficient; however, the efficiency of HR depends on trimming of its excessively long 3′ tails, and is lower than in wild-type bacteria (Ivanković and Đermić 2012; Ivanković *et al.* 2017). HR is not regulated by χ in the *recB1080* mutant (Jockovich and Myers 2001).

An *E. coli* mutant lacking RecA protein is HR-deficient and is therefore unable to repair DSBs, which is reflected in its extreme sensitivity to various genotoxic agents and reduced viability (∼60% of the wild-type) (Kuzminov 1999). This relatively substantial decrease in viability is due to the exonuclease (ExoV) activity of the RecBCD enzyme (Miranda and Kuzminov 2003), which is actually unregulated, or “reckless”, in a *recA* mutant (Willets and Clark 1969). In this mutant, RecBCD degrades DNA duplexes with free dsDNA ends (either damaged or linear molecules introduced exogenously) so heavily that a fraction of the *recA* mutant population is devoid of a chromosome due to its complete degradation (Capaldo and Barbour 1975; Skarstad and Boye 1993).

In this study, we have characterized processing of DSBs introduced synchronously into a radioactively-labeled *E. coli* chromosome by γ-irradiation. Ionizing radiation, such as γ-rays, induce DSBs in DNA by either directly transferring energy to it or indirectly, by creating reactive oxygen species in the cell’s cytoplasm that then damage DNA; during repair of these clustered lesions, DSBs are produced (Bresler *et al.* 1979; Wallace 1998). DSB processing was assessed by measuring degradation of the fragmented chromosome. We show that DNA degradation in γ-irradiated *E. coli* is inhibited by RecA protein concentration and its ssDNA binding affinity. In fact, we show that binding of RecA to ssDNA is sufficient to protect the DNA duplex from degradation. However, DNA degradation as well as its RecA dependence was diminished in cells with preserved 3′ overhangs. Our results suggest that 3′ overhangs exert their influence on DSB processing by inhibiting helicase reloading onto dsDNA ends.

## Materials and Methods

### Bacterial strains, media, growth conditions, phage plating, and microscopy

We used AB1157, a standard HR-, DNA repair-, and DNA degradation-proficient strain (Bachmann 1972). Its derivatives were constructed by P1 transduction, as described earlier (Miller 1992), and are listed in Table 1. Bacteria were grown at 37° in LB medium and on LB agar plates (Miller 1992), supplemented with antibiotics when required.

**Table 1:** Bacterial strains used in this study

Strain	Relevant Genotype	Reference or Construction
AB1157	Wild-type, rec^+^, ExoV^+^	Bachmann (1972)
DE586	Δ*recA774*::*kan*	P1.JW2669 × AB1157 to Kan^r^
DE127	*lexA3* (ind^−^) *malB*::Tn*9*	Laboratory collection
DE583	*recA730* (E38K) *srlD300*::*Tn10 sulA*::Tn*5*	Laboratory collection
DE584	*recA730 sulA*::Tn*5 lexA3 malB*::Tn*9*	P1.DE202 × DE583 to Cm^r^, UV^s^
DE656	Δ*recB1910*::*dhfr*	Laboratory collection
DE657	*recA730 srl*::*Tn10 sulA*::Tn*5* Δ*recB1910*::*dhfr*	P1.DE656 × DE583 to Tm^r^
DE393	*recA_o_281 srlD300*::Tn*10 lexA3 malB*::Tn*9*	Laboratory collection
DE628	*recA_o_281 srlD300*::Tn*10* Δ*malB732*::kan	P1.JW3996 × DE393 to Kan^r^, UV^r^
DE189	Δ*umuDC*::cat	Laboratory collection
DE637	Δ*umuDC*::cat Δ*recA774*::*kan*	P1.DE586 × DE189 to Kan^r^,
DE390	Δ*recD744*::*kan*	P1.JW2787 × AB1157 to Kan^r^
DE595	Δ*recD744*::*kan recA269*::Tn*10*	P1.DE177 × DE390 to Tc^r^
DE101	*recB268*::Tn*10*	Đermić *et al.* (2005)
DE589	*recB268*::Tn*10* Δ*recA774*::*kan*	P1.DE586 × DE101 to Kan^r^
DE590	*recQ1803*::Tn*3*	P1.JJC405 × AB1157 to Ap^r^
DE591	*recQ1803*::Tn*3 recB268*::Tn*10*	P1.DE101 × DE590 to Tc^r^
DE592	*recQ1803*::Tn*3 recB268*::Tn*10* Δ*recA774*::*kan*	P1.DE586 × DE591 to Kan^r^
DE110	Δ*recQ*::*kan*	Ivanković and Đermić (2012)
DE623	Δ*recQ*::*kan recA269*::Tn*10*	P1.DE177 × DE110 to Tc^r^
RIK174	*recBD1080A*	Jockovich and Myers (2001)
DE596	*recB1080* Δ*recA774*::*kan*	P1.DE586 × RIK174 to Kan^r^
DE303	*recJ2052*::Tn*10 kan* Δ*xseA18*::*amp*	Đermić *et al.* (2006a)
DE457	*sbcC201* Δ*exoX769*::*frt* Δ*sbcB780*::*frt*	Laboratory collection
DE585	*sbcC201* Δ*exoX769*::*frt* Δ*sbcB780*::*frt* Δ*xseA758*::*kan*	P1.JW2493 × DE457 to Kan^r^
DE587	*sbcC201* Δ*exoX769*::*frt* Δ*sbcB780*::*frt* Δ*xseA758*::*kan recA269*::Tn*10*	P1.DE177 × DE585 to Tc^r^
DE460	*sbcC201* Δ*exoX769*::*frt* Δ*sbcB780*::*frt* Δ*xseA18*::*amp*	P1.STL4537 × DE457 to Ap^r^
DE630	*sbcC201* Δ*exoX769*::*frt* Δ*sbcB780*::*frt* Δ*xseA18*::*amp* Δ*recD744*::*kan recA269*::Tn*10*	P1.DE177 × DE624 to Tc^r^

Kan, kanamycin; Cm, chloramphenicol; Tm, trimetoprim; Tc, tetracycline; Ap, ampicillin; UV, ultraviolet light.

Exponentially growing bacteria (OD_600_∼0.3) were mixed with serially diluted phage T4 *2* stock and incubated for 15 min at 37°. Soft LB agar was added to each mixture, the mixtures were poured onto LB plates, and incubated for 24 hr at 37°. Determinations were repeated three times.

Bacterial cells (grown identically to those for DNA degradation experiments, except for lacking [^3^H]thymidine) and their chromosomes were visualized in three independent experiments by combined phase-contrast and fluorescent microscopy, as described earlier (Zahradka *et al.* 2009).

### γ-irradiation

Bacteria were exposed to various doses of γ-rays from a ^60^Co source, with a dose rate of ∼2.2 Gy s^−1^. For a bacterial survival assay, bacterial cultures were grown to midexponential phase (OD_600_∼0.3), serially diluted in 67 mM phosphate buffer (pH 7.0), and aliquots spread onto LB plates. The plates were immediately irradiated at room temperature and then incubated at 37° for 24–48 hr.

### Chromosomal DNA degradation

We used the procedure described earlier (Đermić *et al.* 2005, 2006b). The cells were grown overnight in LB medium supplemented with 0.07 MBq ml^−1^ [^3^H]thymidine (specific activity 962 GBq mmol^−1^; Amersham, UK) and 100 µg ml^−1^ deoxyadenosine. Unincorporated [^3^H]thymidine was washed out with phosphate buffer; the cultures were suspended in a double volume of fresh LB medium and divided into two counterparts. One served as an unirradiated control, while the other was irradiated with 400 Gy at 0°. After irradiation, the cultures were incubated at 37° and at intervals duplicate samples were spread onto Whatman filters pretreated with 0.3 M NaOH. The filters were allowed to dry at room temperature, and then were suspended for 30 min in 10% trichloroacetic acid and twice in 5% trichloroacetic acid at 4°. Trichloroacetic acid precipitates high molecular weight DNA, while the low molecular weight DNA is washed away. The filters were then washed in 1:1 solution of ether and ethanol at 4° for 30 min, and then in ether at room temperature. Acid-precipitable radioactivity of the filters represents the amount of the intact chromosomal DNA, and was measured by scintillation counting (Liquid Scintillation Analyzer, Tri-Carb 2810 TR, Perkin Elmer). On average, 1500–3000 cpm was measured in unirradiated samples.

The frequency of genomic DSBs inflicted by γ-irradiation ranges from 0.004 to 0.01 DSBs/Gy/Mbp in various organisms (Daly 2009). The dose of 400 Gy is therefore expected to produce at least eight DSBs per *E. coli* chromosome. This is in accord with a measured rate of DSB induction in the *E. coli* chromosome of 2.7 DSBs per 100 Gy (Ulmer *et al.* 1979) (which would amount to ∼10 DSBs at 400 Gy), and also with the prediction of ∼9 DSBs per cell at 400 Gy (Shee *et al.* 2013), based on direct detection of DSBs *in vivo* using a fluorescent DSB binding protein.

### Preparation of cell-free extracts and western analysis

Bacterial cultures (grown identically as those for DNA degradation experiments, except for lacking [^3^H]thymidine) were split into two parts, one of which was irradiated with 400 Gy at 0°. After irradiation, both cultures were incubated at 37° for an additional 35 min. Cell-free extracts were prepared as previously described (Vujaklija and Maček 2012), with some modifications. Briefly, the cells were harvested from 20 ml of culture by centrifugation at 5000 × *g* and washed with 25 mM Tris-HCl pH 8.3, 1 mM NaCl, 1 mM EDTA, 1 mM DTT, and 0.5 mM PMSF. The cells were suspended in the same buffer and disrupted by sonication. Cell debris was removed by centrifugation at 12000 × *g* and the supernatant was used as a cell-free extract. Next, 15 µg of total protein were loaded into each lane and resolved by SDS-PAGE under reducing conditions in 10% gels. After electrophoresis, the separated proteins were transferred to an Amersham Hybond-P PVDF membrane (GE Healthcare). The membrane was stained with Amido Black (Sigma Aldrich, St. Louis) for protein visualization. This was a loading control and allowed determination of protein transfer efficiency across the blot. Western analysis performed as described (Vujaklija and Maček 2012) was used to estimate the relative concentrations of RecA protein in each sample. RecA was detected on western blots with the polyclonal anti-RecA antibody ab63797 (Abcam) diluted 1:6000. Antibody binding was visualized with peroxidase-coupled goat anti-rabbit antibody diluted 1:30,000 and Amersham ECLTM Western Blotting Reagent Pack (GE Healthcare). The specificity of the antibody was examined using an *E. coli* strain lacking the *recA* gene. Two biological replicates of each strain were analyzed by immunoblotting. ImageJ, a Java-based image processing program (Schneider *et al.* 2012), was applied to analyze western blot signal intensity.

### Data availability

Strains are available upon request. All the data necessary for confirming the conclusions presented in this paper are represented in the paper.

## Results

Synchronous DSBs were induced into *E. coli* DNA by γ-irradiation and their processing was monitored by measuring degradation of the fragmented chromosome, which was labeled with [^3^H]thymidine.

### RecA inhibits DNA degradation in γ-irradiated E. coli

**Figure 1:**
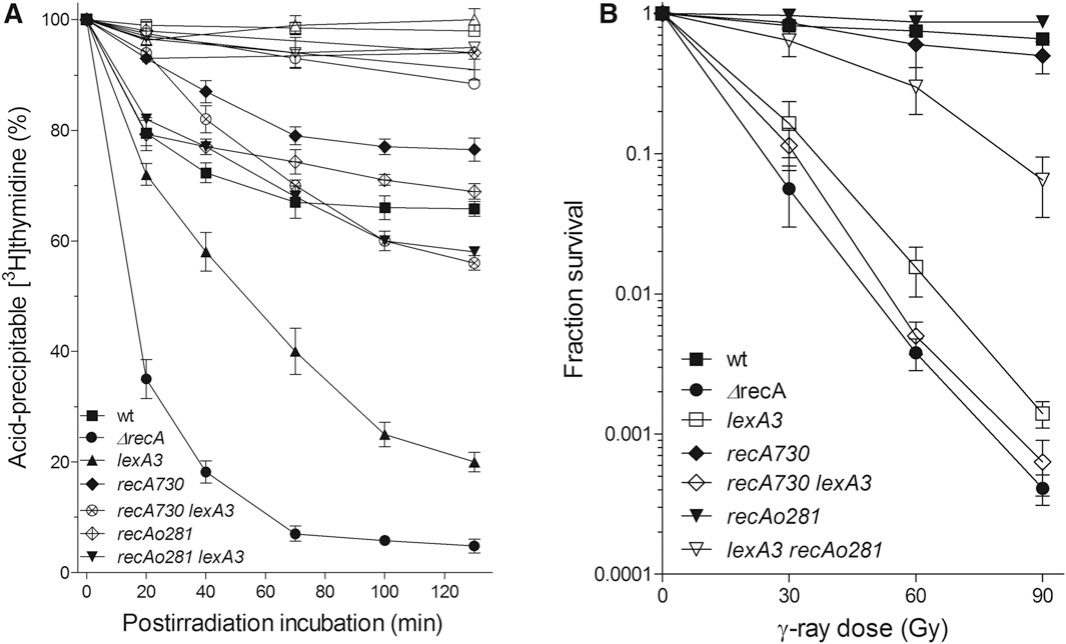
(A) DNA degradation in γ-irradiated *E. coli* depends on RecA protein concentration and its rate of association with ssDNA. Bacterial cultures were divided into two counterparts; one served as a control (open symbols), while the other was irradiated with 400 Gy (closed symbols). The cultures contained [^3^H]thymidine-labeled chromosome; kinetics of their degradation were monitored during incubation at 37°. AB1157 (□,▪); *∆recA* (○,●); *lexA3* (▵,▴); *recAo281* (+,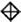); *lexA3 recAo281* (▿,▾); *recA730* (◊,♦); and *recA730 lexA3* (×,⊗). Each value is a mean of three independent experiments, with error bars representing SD. (B) Survival of γ-irradiated bacteria. Wild-type strain AB1157 (▪) and its *recA* (●), *lexA3* (h), recA_o_281 (▾), *lexA3 recA_o_281* (▿), *recA730* (♦), and *recA730 lexA3* (◊) derivatives. Fraction survival is given as a fraction of the unirradiated control. Each value is a mean of three independent experiments, with error bars representing SD. ssDNA, single-stranded DNA; wt, wild-type.

Figure 1A shows a time course of chromosomal DNA degradation in *E. coli* irradiated with 400 Gy of γ-rays. Irradiated wild-type strain AB1157 degraded ∼30% of its DNA during ∼60 min, after which degradation ceased and the remaining DNA was stable for another ∼60 min of postirradiation incubation at 37° (Figure 1A). This result is in accord with previous studies (Đermić *et al.* 2005, 2006b).

On the other hand, a *recA* null mutant (DE586) showed much stronger, continuous DNA degradation that resulted in degradation of ∼95% of its cellular DNA after ∼120 min postirradiation incubation (Figure 1A). Therefore, we confirmed a “reckless” DNA degradation in the *recA* mutant.

**Figure 2:**
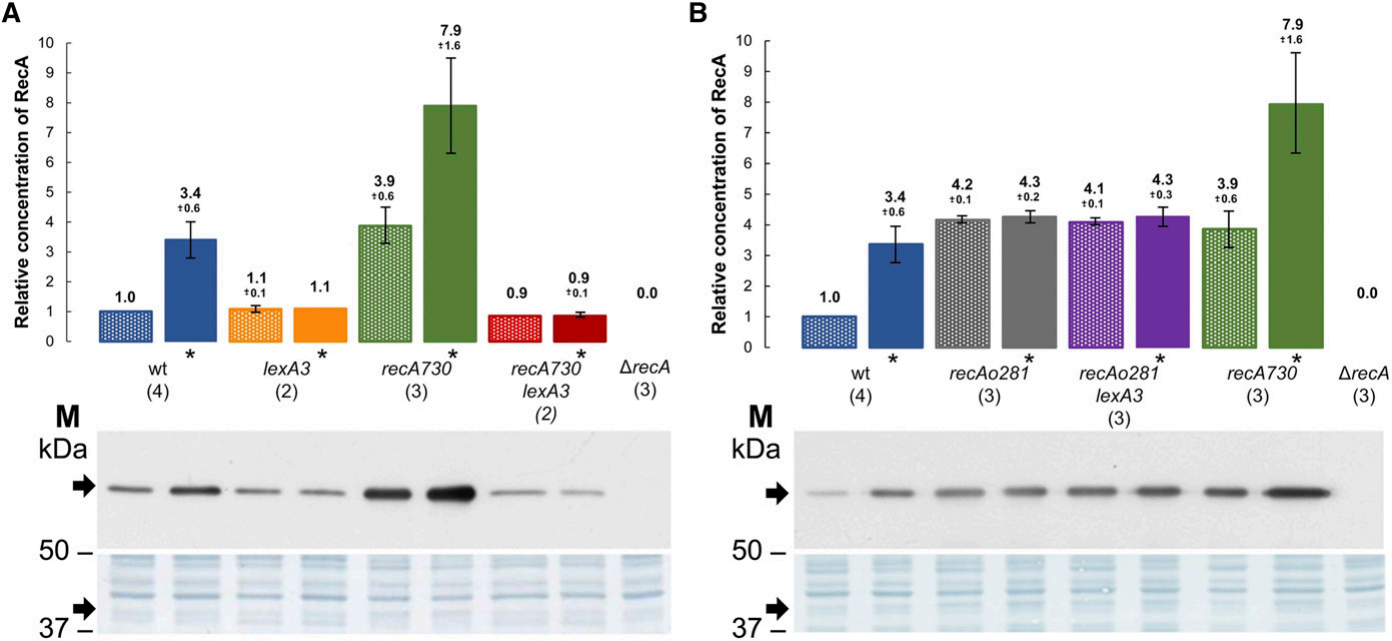
Immunoblotting analysis of relative RecA protein concentrations in *E. coli* (A and B). Protein samples obtained from the cells that were irradiated with 400 Gy of γ-rays and subsequently incubated for 35 min at 37° are marked with *. PVDF membranes stained with Amido Black were used as a loading control (lower panels). SDS-PAGE gels and PVDF membranes are from one experiment, while graph depicts mean and SD (error bars) from multiple replicates (numbered in parentheses) from two independent experiments. PVDF, polyvinylidene fluoride; SDS-PAGE, sodium dodecyl sulfate polyacrylamide gel electrophoresis; wt, wild-type.

Since RecA protein regulates SOS induction and is itself regulated by SOS, we wanted to assess the effect of the SOS response on DNA degradation in γ-irradiated *E. coli*. For that, we made the SOS system in AB1157 uninducible by introducing a *lexA3* allele, coding for a noncleavable SOS repressor. By western analysis, we estimated the relative concentrations of RecA protein in the wild-type cells that were or were not exposed to γ-rays. Our result revealed an increase of around threefold the basal RecA concentration in the irradiated wild-type strain (35 min after irradiation) compared to the unirradiated control (Figure 2). The lower level of SOS induction compared to the previous study (Sassanfar and Roberts 1990) is likely due to short postirradiation incubation time and different SOS-inducing agents used. As expected, no change of (basal) RecA concentration was observed in the *lexA3* mutant upon irradiation (Figure 2A). As shown in Figure 1A, the *lexA3* mutant also showed continuous DNA degradation during ∼120 min postirradiation incubation, but the amount of degradation was lower than in the *recA* mutant, with ∼20% of its DNA being spared. This result shows that the SOS system influences DNA degradation in γ-irradiated *E. coli*. In order to check which function of the SOS is important for suppression of DNA degradation, we made use of *recA_o_281*, an operator mutation that enables SOS-independent constitutive overexpression of RecA protein (Clark 1982). RecA concentration was ∼4 fold higher in a *recA_o_281* mutant compared to the unirradiated wild-type strain, while being about the same in their irradiated counterparts (Figure 2B). DNA degradation was similar in the *recA_o_281* mutant and wild-type strain (Figure 1A). A *lexA3 recA_o_281* mutant is SOS-deficient, yet expresses similarly elevated (around fourfold) concentration of RecA protein (Figure 2B). After 2 hr of postirradiation incubation, it degraded ∼40% of its DNA, which is similar to degradation in the wild-type strain, whereas about threefold more of its DNA was preserved compared to DNA in the irradiated single *lexA3* mutant (Figure 1A). This suggests that RecA concentration is important for suppressing DNA degradation, while other parts of the SOS response have a minor effect. Therefore, our results reveal an inverse relationship between RecA concentration and DNA degradation in irradiated *E. coli*, which led us to conclude that RecA protein suppresses DNA degradation by mass effect.

Next, we wanted to determine how the RecA rate of association with ssDNA influences DNA degradation in irradiated cells. For that, we used a *recA730* mutant, which produces the RecAE38K mutant protein that shows an increased rate of association with ssDNA and hence competes more efficiently with SSB protein for binding to ssDNA (Lavery and Kowalczykowski 1992). When introduced into AB1157, the *recA730* mutation reduced DNA degradation in the resulting strain ∼1.5-fold (Figure 1A). This effect can be caused by an increased affinity of the RecAE38K for ssDNA, but also by an increased concentration of the enzyme in that mutant due to constitutive SOS induction. RecAE38K concentration in unirradiated and irradiated cells of the *recA730* mutant was around four- and eightfold higher, respectively, than the RecA concentration in an unirradiated wild-type strain (Figure 2). We measured the effect of the increased affinity of RecAE38K for ssDNA by using a *lexA3 recA730* mutant, which produces a basal level of RecA E38K that is equal to that of RecA in the *lexA3* mutant (Figure 2A). The *lexA3 recA730* mutant showed greatly reduced (about twofold) DNA degradation compared to that in the *lexA3* mutant (Figure 1A), revealing that RecA dependency of DNA degradation is proportionate to RecA’s rate of association with ssDNA. In order to check whether the protective effect of RecAE38K protein on DNA degradation is indeed due to its high affinity to ssDNA, and not to its possible nonspecific binding to dsDNA, we determined the titer of T4 *2* phage in *recA730* and *recA730 recB* mutants. The T4 *2* mutant phage genome is a linear dsDNA with free, blunt ends and hence susceptible to RecBCD binding and nucleolytic degradation. If RecAE38K would bind to dsDNA, hence blocking the access and activity of RecBCD on it, the titer of T4 *2* phage should increase. In comparison to a *recB* mutant (which cannot degrade the T4 *2* phage genome and therefore enables maximal phage yield), the T4 *2* phage plating efficiencies in the *recA730* and wild-type strains were 0.0019 ± 0.001 and 0.0017 ± 0.0007, respectively, which is significantly different (*P* = 0.001, two-tailed *t*-test) from the *recB* derivative of *recA730* mutant that had 0.94 ± 0.19 of *recB’s* titer (*n* = 3), suggesting that RecBCD inhibits phage propagation in the *recA730* mutant and that phage genome metabolism is otherwise not impaired in that mutant. Hence, we infer that RecAE38K protein does not interfere with degradation of purely dsDNA.

The difference in degradation between *recA730* and *recA730 lexA3* mutants (Figure 1A) is likely mostly due to different concentrations of the RecAE38K protein in these strains. The same, moderate amount of DNA degradation observed in the *lexA3 recA730* and *lexA3 recA_o_281* mutants (Figure 1A) indicates that, for DNA degradation inhibition, the higher RecA rate of association with ssDNA can compensate for lower RecA concentration, and that SOS induction is not required *per se* for protection of cellular DNA from degradation.

In summary, we have shown that RecA protein protects a fragmented chromosome from degradation in γ-irradiated wild-type *E. coli*. The protective activity depends on RecA concentration and its rate of association with ssDNA.

### DNA degradation is distributed uniformly in wild-type cells

**Figure 3:**
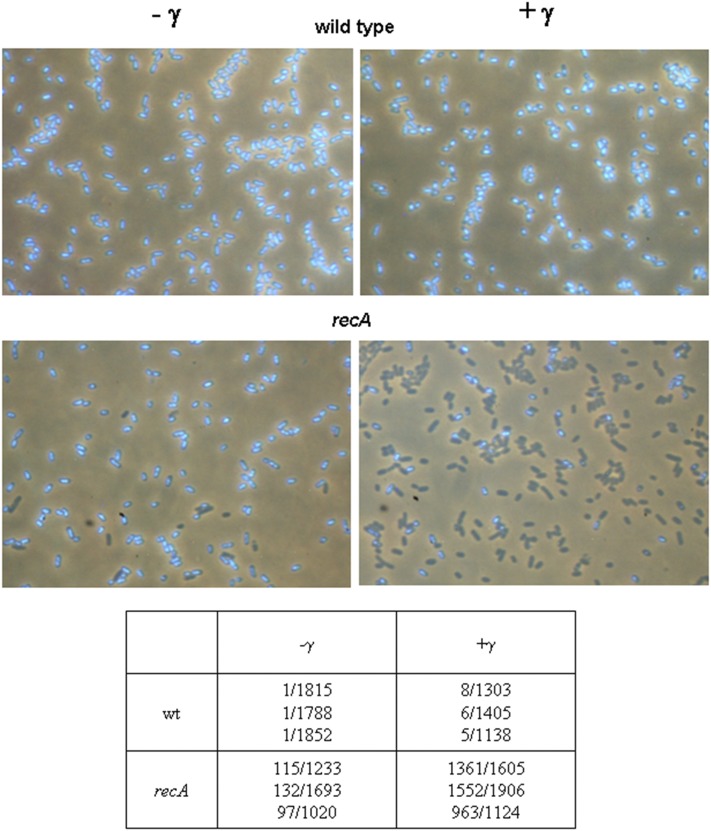
DNA degradation is strictly controlled in wild-type, but not in *recA* cells. A fraction of anucleate cells was determined in wild-type (AB1157) and *recA* populations, which were either unirradiated (left side of the panel) or irradiated with 400 Gy of γ-rays and then incubated for 90 min at 37° (right side of the panel). Cells were fixed with osmium tetroxide, their DNA stained with DAPI, and visualized by fluorescence microscopy. An anucleated cell count in an analyzed population from three experiments is represented in a table. DAPI, 4’,6-diamidino-2-phenylindole; wt, wild-type.

The DNA degradation experiments used in this study provide bulk population data that do not give us information on degradation distribution in a population. To assess the distribution of DNA degradation in a cell population, we visualized bacteria by microscopy and determined the fraction of cells lacking DAPI staining material in a population of wild-type (AB1157) and *recA* bacteria after 90 min postirradiation incubation. Unirradiated populations of wild-type and *recA* cells contained 0.055 ± 0.001% and 8.87 ± 0.93% anucleate cells, respectively (Figure 3). On the other hand, wild-type and *recA* populations γ-irradiated with 400 Gy had 0.49 ± 0.10% and 84.1 ± 2.4% of cells lacking a DAPI signal, respectively, after 90 min incubation (Figure 3). These data suggest that DNA degradation in wild-type cells is tightly regulated since it is very uniform, so that ∼35% of their degraded cellular DNA (Figure 1A) is reflected in <1% of chromosomeless cells. Conversely, only ∼16% of irradiated *recA* cells contained DNA, which is consistent with heavy, uncontrolled DNA degradation in them, sparing just ∼5% of their DNA (Figure 1A).

### Role of RecA protein in survival of γ-irradiated cells

Since degradation of a fragmented chromosome reflects DSB processing reactions, we wanted to relate it to a DSB repair process. Therefore, we determined survival of γ-irradiated bacteria as a measure of the efficiency of their DSB repair. As expected, the *recA* mutant showed an extreme sensitivity to γ-rays; at 90 Gy dose, its survival was more than three orders of magnitude lower than that of the wild-type strain AB1157 (Figure 1B). The *lexA3* mutant had somewhat higher survival than the *recA* mutant (Figure 1B), which is not surprising considering that it retains basal RecA concentration. Survival of the *lexA3 recA_o_281* mutant was considerably higher than that of the *lexA3* mutant (also due to higher RecA concentration), whereas the *recA_o_281* mutant had the same survival as the wild-type strain (Figure 1B).

While the *recA730* mutant had essentially the same survival as the wild-type strain, its *lexA3* derivative showed extreme sensitivity to γ-rays, similar to that of the *recA* and *lexA3* mutants (Figure 1B). Since the *recA730 lexA3* mutant showed highly reduced DNA degradation compared to the *recA* (and *lexA3)* mutant, whereas it displayed almost identical γ-survival (compare Figure 1, A and B), we conclude that reducing “reckless” degradation is insufficient to enable DNA repair.

A comparison of γ-survivals shows that the important factors for higher survival of γ-irradiated cells are: higher RecA protein concentration (hence the higher survival of *recA_o_281 lexA3* than *lexA3* and *recA730 lexA3* mutants) and SOS induction (causing increased survival of *recA_o_281* compared to the *recAo281 lexA3* mutant).

### RecA inhibition of DNA degradation is independent of DNA polymerase V

**Figure 4:**
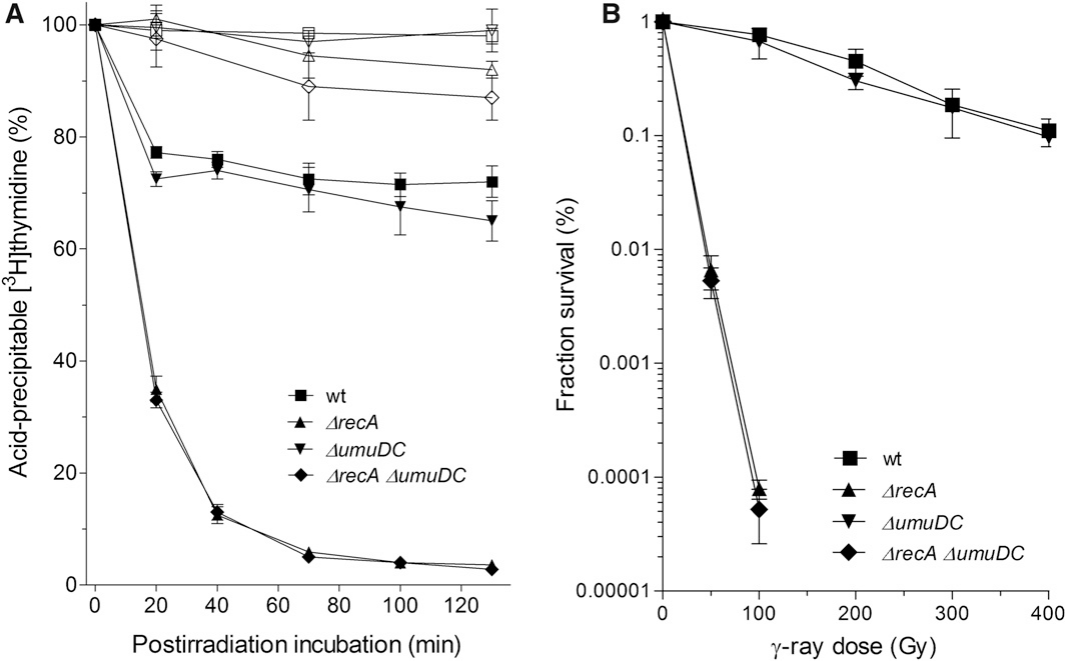
DNA degradation is unrelated to DNA polymerase V. Kinetics of [^3^H]thymidine-labeled DNA degradation (A), and survival (B) of γ-irradiated wild-type strain AB1157 (▪) and its *umuDC* (▾), *recA* (▴), and *umuDC recA* (♦) derivatives. For DNA degradation assay, bacterial cultures were divided into two fractions; one served as a control (open symbols), while the other was irradiated with 400 Gy (closed symbols). Fraction survival is given as the fraction of the unirradiated control. Each value is a mean of three independent experiments, with error bars representing SD. wt, wild-type.

In *E. coli*, RecA is required for three processes, namely for HR, SOS induction, and activation of mutagenic DNA polymerase V (PolV) (see *Introduction*). Since we have shown that RecA’s function in inhibition of DNA degradation does not necessarily correspond to its role in DSB repair and SOS induction, we wanted to determine its relationship with PolV activation. For that goal, we determined DNA degradation and γ-survival of a PolV-deficient *umuDC* mutant and its *recA* derivative. As shown in Figure 4, the PolV-deficient mutant had mildly increased DNA degradation compared to the wild-type strain, while its γ-survival was the same as that of the wild-type strain. Furthermore, no difference was observed in DNA degradation and survival of *umuDC recA* and *recA* mutants (Figure 4). These results indicate that the RecA protective role during DNA degradation does not depend on PolV.

### DNA degradation in recB and recD mutants is inhibited by RecA

**Figure 5:**
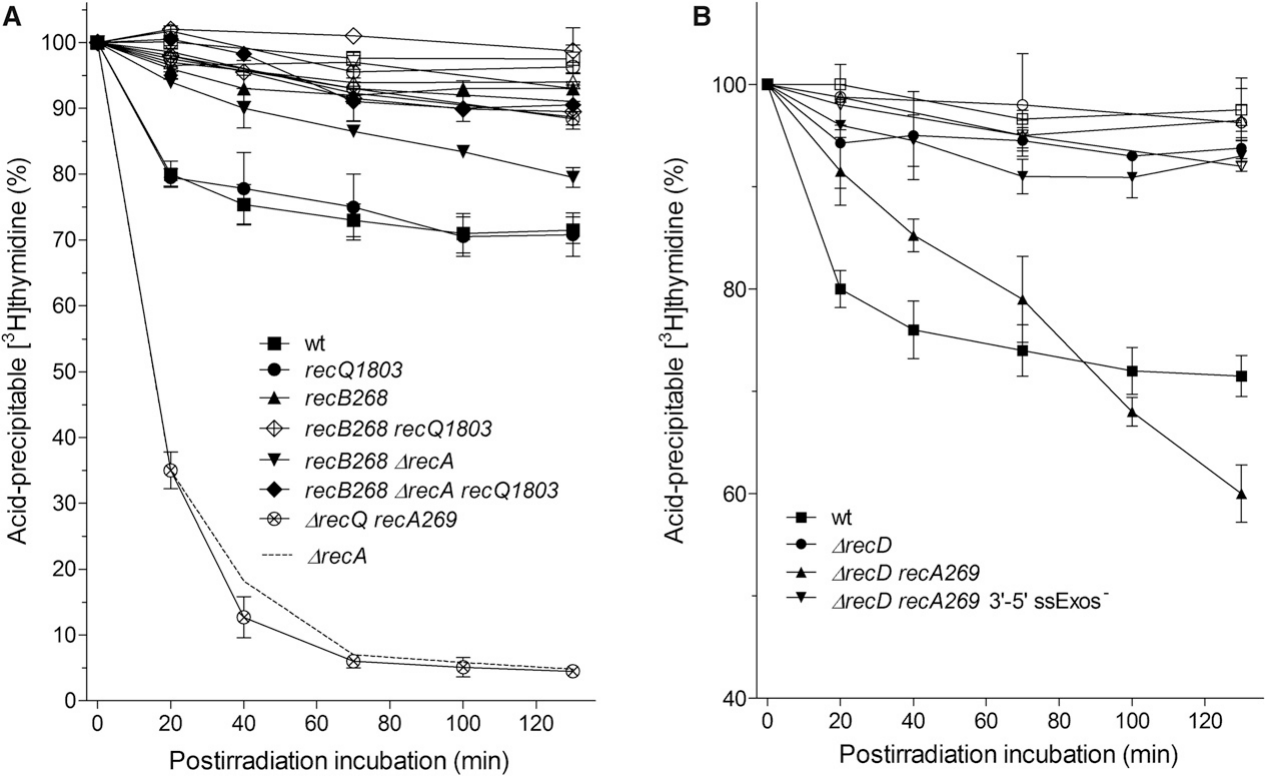
DNA degradation in *recB* (A) and *recD* (B) mutants is inhibited by RecA protein. Bacterial cultures were divided into two counterparts; one served as a control (open symbols), while the other was irradiated with 400 Gy (closed symbols). The cultures contained [^3^H]thymidine-labeled chromosome; kinetics of its degradation was monitored during incubation at 37°. (A) AB1157 (□,▪); *recQ* (○,●); *recB* (▵,▴); *recB recQ* (+,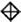); *recB recA* (▿,▾); *recB recA recQ* (◊,♦); *recQ recA* (×,⊗), and *recA* (- - -). (B) AB1157 (□,▪); *recD* (○,●); *recD recA* (▵,▴); and ExoI^−^ ExoVII^−^ ExoX^−^ SbcCD^−^ RecA^−^ RecD^−^ (▿,▾). Each value is a mean of three independent experiments, with error bars representing SD. wt, wild-type.

We wanted to determine whether the RecA inhibition of DNA degradation is restricted to RecBCD-catalyzed reactions, or whether it is a more general phenomenon. For this aim, we determined DNA degradation in γ-irradiated *recB* and *recD* mutants. No degradation was observed in these mutants (Figure 5, A and B), which is not surprising considering their ExoV^−^ phenotype. However, their *recA* derivatives did degrade their damaged DNA. A *recB recA* mutant degraded its DNA continuously during postirradiation incubation, with degradation reaching a level close to that of the wild-type strain after 130 min (Figure 5A). This result indicates that RecA protects DNA from degradation in a *recB* null mutant. Since in the *recB* mutant dsDNA ends are processed by the RecQ helicase, we evaluated the role of RecQ in DNA degradation in the *recB recA* mutant. A triple *recB recA recQ* mutant degraded ∼10% of its DNA after 130 min incubation, which is about twofold less compared to its parental RecQ^+^ strain (Figure 5A), indicating that a helicase activity participates in DSB processing reactions that result in degradation of a shattered *E. coli* chromosome. At the same time, the *recQ* mutation did not affect DNA degradation in wild-type or *recA* and *recB* null mutants (Figure 5A), suggesting that the RecQ helicase role is specifically pronounced in the *recB recA* mutant.

A *recD recA* mutant (DE595) also showed constitutive DNA degradation; ∼40% of its DNA was degraded after 2 hr of incubation, thus exceeding the degradation level observed in the wild-type strain (Figure 5B). The RecBC enzyme is a nuclease-free helicase whose unwound products are subject to the activity of ssExos (Đermić 2006; Đermić *et al.* 2006a; Rinken *et al.* 1992), which results in limited degradation of the unwound DNA (Rinken *et al.* 1992). An earlier study (Kuzminov and Stahl 1997) has shown degradation of linearized plasmid DNA in a *recD recA* but not in a *recB recA* mutant.

In summary, we have shown that the RecA inhibition of DNA degradation is not only restricted to RecBCD-expressing wild-type bacteria, but also applies to the *recB* and *recD* mutants, suggesting that it is a more general phenomenon in *E. coli*. Also, we have shown that RecA-controlled DNA degradation involves a helicase activity.

### DNA degradation in the recB1080 mutant is not inhibited by RecA protein

**Figure 6:**
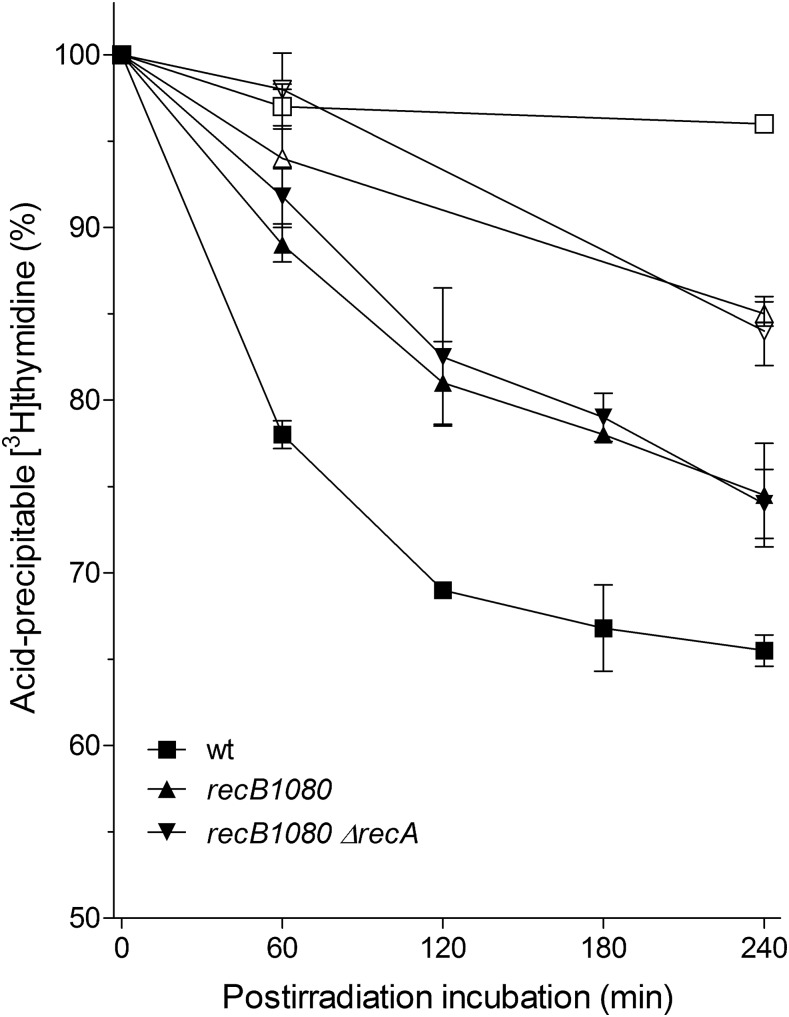
DNA degradation in the *recB1080* mutant is weak and uninhibited by RecA protein. Bacterial cultures were divided into two counterparts; one served as a control (open symbols), while the other was irradiated with 400 Gy (closed symbols). The cultures contained [^3^H]thymidine-labeled chromosome; kinetics of its degradation was monitored during incubation at 37°. AB1157 (□,▪); *recB1080* (▵,▴); and *recB1080 recA* (▿,▾). Each value is a mean of three independent experiments, with error bars representing SD. wt, wild-type.

To further characterize the interplay of a helicase activity and RecA polymerization in DNA degradation, we made use of a *recB1080* mutant strain RIK174, which produces the RecB^1080^CD enzyme. This enzyme is a fast and processive helicase, but is also nuclease-free and unable to load RecA onto the unwound 3′ tail (Yu *et al.* 1998; Anderson *et al.* 1999). *In vitro*, the enzyme unwinds the linear DNA duplex, releasing full length, RecA-free ss tails (Yu *et al.* 1998; Anderson *et al.* 1999). γ-irradiated RIK174 degraded ∼25% of its DNA after 240 min of incubation, which was ∼70% of DNA degradation in the wild-type bacteria (Figure 6). However, a *recB1080 recA* mutant showed a similarly low level of DNA degradation as its RecA^+^ parental strain (Figure 6), suggesting that DNA degradation in the *recB1080* mutant is not inhibited by the RecA protein. This result indicates that, during processing of DSBs by a powerful nuclease-free helicase, long unwound tails prevent excessive DNA degradation, in effect relieving the RecA regulation of DNA degradation.

### Inactivation of 3′–5′ ssExos greatly reduces DNA degradation and makes it independent of RecA

**Figure 7:**
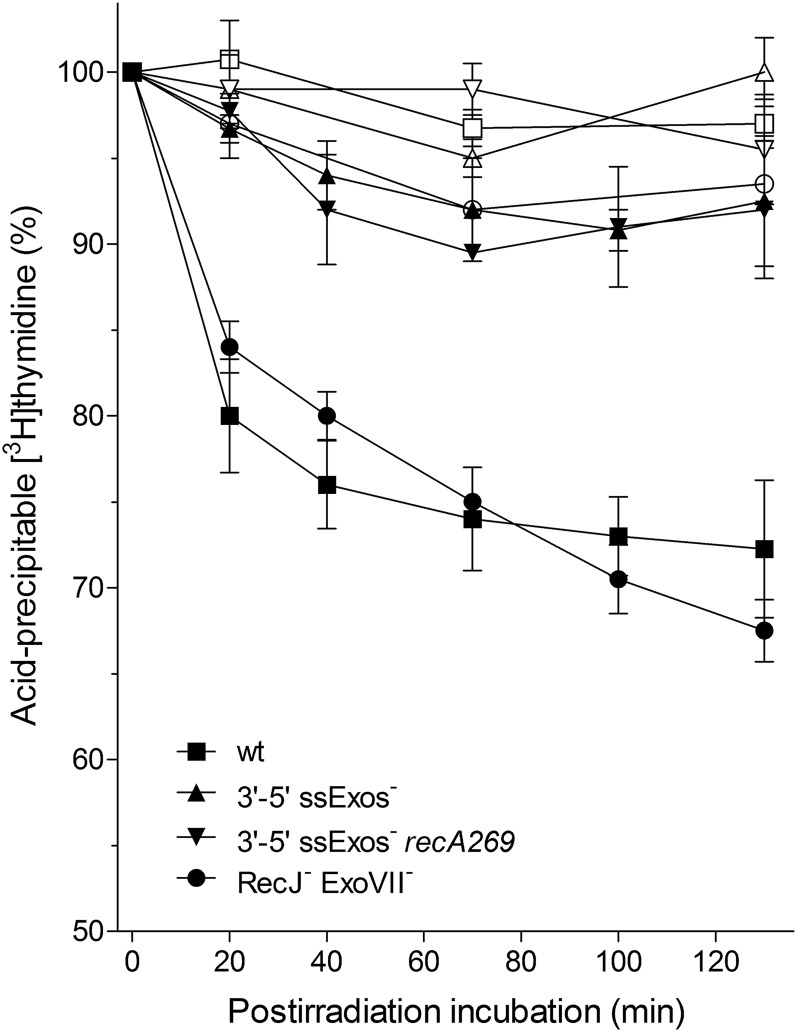
DNA degradation in a mutant lacking 3′–5′ ssExos is very weak and not inhibited by RecA protein. Bacterial cultures were divided into two fractions; one served as a control (open symbols) while the other was irradiated with 400 Gy (closed symbols). The cultures contained [^3^H]thymidine-labeled chromosome; kinetics of its degradation were monitored during incubation at 37°. AB1157 (□,▪); ExoI^−^ ExoVII^−^ ExoX^−^ SbcCD^−^ (▵,▴); ExoI^−^ ExoVII^−^ ExoX^−^ SbcCD^−^ RecA^−^ (▿,▾); and RecJ^−^ ExoVII^−^ (○,●). Each value is a mean of three independent experiments, with error bars representing SD. wt, wild-type.

It was shown earlier that inactivation of ExoI, SbcCD, and ExoVII, ssExos that degrade 3′ overhangs, prevents “reckless” DNA degradation in a *recA* mutant (Zahradka *et al.* 2009; Repar *et al.* 2013). We made a quadruple mutant deficient in ExoI, SbcCD, ExoVII, and Exonuclease X (ExoX), ssExos that trim 3′ overhangs (Lovett 2011), and measured its DNA degradation. The mutant degraded ∼10% of its DNA after 130 min of incubation (Figure 7), which is close to the amount of DNA degraded in its unirradiated part (Figure 7), and is greatly reduced compared to degradation in its ssExo^+^ parental strain. On the other hand, DNA degradation in a strain lacking 5′–3′ ssExos RecJ and ExoVII (Lovett 2011) was similar to that in wild-type bacteria amounting to 31 ± 4% of genomic DNA after 130 min of incubation (Figure 7).

Since the mutant lacking four 3′–5′ ssExos is an ExoV^−^ phenocopy with respect to DNA degradation, although it expresses an intact RecBCD enzyme, we checked the enzyme’s nuclease activity by assessing its ability to inhibit growth of a T4 *2* phage. Relative to the titer on the *recB* mutant, T4 *2* phage plating efficiency on the wild-type strain was 0.0067 ± 0.0043, and that on the 3′–5′ ssExos-deficient mutant was 0.019 ± 0.012 (*n* = 3), which is not significantly different (*P* = 0.170, two-tailed *t*-test). This result indicates that RecBCD is proficient in binding to and degrading phage DNA in the mutant lacking 3′–5′ ssExos.

A *recA* derivative of the quadruple ExoI^−^ SbcCD^−^ ExoVII^−^ ExoX^−^ mutant showed about the same, low-level ∼10% degradation as its RecA^+^ counterpart (Figure 7), suggesting that DSB processing in bacteria with a preserved 3′ tail is not affected by RecA.

Similarly, DNA degradation in a *recD recA* mutant was greatly abolished by inactivation of the four 3′–5′ ssExos (Figure 5B). Therefore, we conclude that the constitutive chromosome degradation observed in the irradiated *recD recA* mutant is dependent on degradation of 3′-ended ss overhangs by the 3′–5′ ssExos.

Hence, our results indicate that 3′ overhangs suppress DSB processing, especially when spared from 3′–5′ ssExos.

## Discussion

To gain better insight into the *in vivo* processing of DSBs, we followed the degradation of radioactively-labeled chromosomal DNA of γ-irradiated *E. coli*. DSBs in that bacterium are repaired by the RecBCD enzyme, which upon binding to a (nearly blunt) dsDNA end unwinds the DNA duplex and degrades both of the unwound strands. Only after interaction with the χ sequence does the enzyme starts a resection process, meaning that it continues its degradation of the 5′ strand while ceasing trimming of the 3′ strand. Therefore, DNA degradation is an essential and indivisible part of DSB processing in *E. coli* (there is no situation in which RecBCD activity on DNA is nuclease-free), and by assessing DNA degradation one can indeed get an insight into DSB processing. RecBCD enzyme is the strongest DNase in *E. coli* (Kuzminov 1999), yet its nuclease activity is augmented in bacteria lacking the RecA protein, wherein it becomes unregulated (“reckless”), leading to complete degradation of a chromosome (Capaldo and Barbour 1975; Skarstad and Boye 1993). “Reckless” degradation in *recA* null mutants is attributed to either impaired χ regulation of the RecBCD enzyme (Kuzminov *et al.* 1994; Kuzminov and Stahl 1997) or to a lack of RecA protection of the frayed ends of a processed DNA molecule (Dabert *et al.* 1992). We have shown here that recruitment of RecA onto ssDNA inhibits degradation at DSBs in wild-type, *recB*, and *recD* genetic backgrounds, indicating that the RecA protection of DNA from degradation is a general phenomenon in *E. coli*. Since there is no χ activity in the *recB* and *recD* mutants, and yet degradation of their DNA is still inhibited by RecA, we infer that the physical protection by RecA binding on (3′-terminated) ss overhangs is the main mechanism for RecA inhibition of DNA degradation. A recent study indicated that a RecA-ssDNA complex is resistant to degradation by nucleases (Kohiyama *et al.* 2013). Analogously, a human RecA ortholog, RAD51 recombinase, prevents excessive DNA nucleolytic degradation in UV-irradiated human cells (Vallerga *et al.* 2015), indicating conservation of the inhibitory role of recombinase proteins in DNA degradation, thus signifying its importance.

However, our results do not exclude the possibility that RecBCD’s χ activity is indeed impaired in the *recA* mutant, nor do they determine the possible contribution of each of the two mechanisms to DNA protection in that mutant.

Interestingly, our results show that the ability of the RecA protein to protect DNA from degradation differs from its role in DSB repair. The *recA730 lexA3* mutant has greatly suppressed DNA degradation compared with a *lexA3* mutant [both contain about the same concentration of RecA(E38K) protein], while having similar γ-survival. This phenotype is not unique as the *recB* mutant has it too; it also has RecA-inhibited DNA degradation and extremely poor survival. Thus, our results suggest that the RecA role in DNA degradation regulation is less challenging than its role in DSB repair; the former is about preventing 3′–5′ ssExos from degrading the 3′ tail (for which, binding of a couple of RecA molecules would likely suffice), while the latter requires production of a functional RecA nucleofilament. Furthermore, *recB* and *recD* null mutants share poor DNA degradation (suppressed by RecA), while the former is deficient for DSB repair and the latter is proficient. Also, *recA730 lexA3* and *recA_o_281 lexA3* mutants have about the same amount of DNA degradation, while their γ-survivals greatly differ.

**Figure 8:**
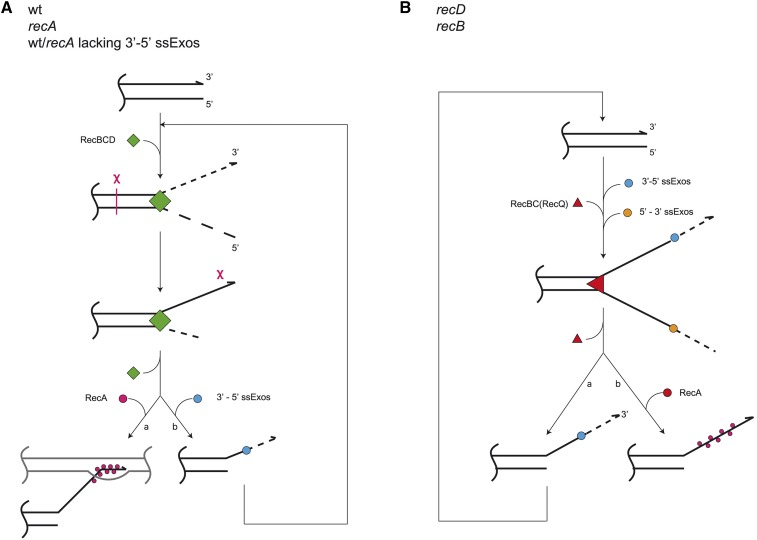
Processing of a dsDNA end in several *E. coli* genetic backgrounds. (A) In wt bacteria, dsDNA processing is initiated by binding of RecBCD enzyme onto it, which unwinds the DNA duplex while degrading both unwound strands. Upon interaction with the χ site, the enzyme stops the 3′ strand degradation, while continuing DNA unwinding and degradation of the 5′ strand. χ-modified RecBCD also facilitates RecA loading onto a post-χ 3′ overhang (a). In the *recA* null mutant, RecBCD degrades both unwound strands before eventually being released from DNA, which might be independent of χ, leaving a protruding 3′ tail. The 3′ tail prevents subsequent reloading of RecBCD onto a processed dsDNA end unless/until 3′–5′ ssExos degrade it (b). Since both RecA^+^ and RecA^−^ cells have similar, low DNA degradation when 3′–5′ ssExos are inactive [blocked (b) path], these exonucleases apparently act on a post-χ 3′ tail in wt cells [hence path (b) may apply to wt cells too]. (B) In *recA* derivatives of *recB* and *recD* mutants, dsDNA end processing starts with RecQ and RecBC helicase unwinding of the DNA duplex, respectively. The unwound 3′- and 5′-terminated strands are degraded by 3′–5′ and 5′–3′ ssExos, respectively. The degradation of the 5′ strand is apparently faster than that of its complementary 3′ strand (otherwise *recD* mutant would not be HR proficient) and thus the latter remains after helicase detachment from DNA. The protruding 3′ tail is then degraded by 3′–5′ ssExos (a), therefore enabling creation of a (nearly) blunt DNA end and hence repeated loading of RecQ or RecBC helicase. In RecA^+^ variants of *recB* and *recD*, RecA binds to the 3′ tail (b) and prevents RecQ reloading onto the DNA end in the *recB* mutant, while blocking its trimming and recreation of the blunt DNA end in the *recD* mutant and hence inhibits RecBC reloading. dsDNA, double-stranded DNA; ssExos, exonucleases that degrade a 3′ tail; wt, wild-type.

However, we revealed two notable exceptions in the RecA-imposed control of DNA degradation, which enabled further insight into the mechanism of DSB processing regulation in *E. coli*. Namely, inactivation of four ssExos (ExoI, ExoVII, ExoX, and SbcCD) that degrade a 3′ tail (Lovett 2011) greatly reduced DNA degradation in both RecA^+^ and RecA^−^ bacteria, making them an ExoV^−^ phenocopy, even though the RecBCD enzyme is active in these cells and able to degrade DNA (as it prevented proliferation of the T4 *2* phage, whose genome is blunt-ended). Since RecBCD poorly degrades fragmented chromosomes in cells lacking 3′–5′ ssExos while retaining ExoV activity, we infer that the enzyme is affected in binding to DNA, *i.e.*, creation of blunt DNA ends is suppressed. A previous study showed that a strain lacking three 3′–5′ ssExos (ExoI, ExoVII, and SbcCD) retains nearly wt capacity for DNA repair, whereas its *recA* derivative had inhibited DNA degradation (ExoV^−^), thus indicating that these ssExos are not required for blunting the initial irradiation-produced dsDNA ends although being critical for “reckless” DNA degradation (Repar *et al.* 2013). Because 3′–5′ ssExos inactivation is epistatic to RecA deficiency for DNA degradation, we conclude that RecA inhibits DNA degradation by preventing trimming of 3′-terminated overhangs by 3′–5′ ssExos; hence, when these ssExos are absent, RecA becomes dispensable. Therefore, in cells lacking 3′–5′ ssExos, DSB processing is inhibited by preserved 3′ overhangs, irrespective of RecA protein (Figure 8A). Conversely, RecA deficiency is epistatic to 3′–5′ ssExos deficiency for DNA repair, thus additionally emphasizing differences in roles of the RecA protein in DNA repair and DNA degradation in *E. coli*.

Another situation where DNA degradation is unaffected by RecA concerns the *recB1080* mutant, whose *recA* derivative had about the same, low level of DNA degradation as its parental RecA^+^ strain. A combination of fast and processive helicase activity and lack of nuclease and RecA loading activities of RecB^1080^CD enzyme likely results in long, RecA-free overhangs in the *recB1080* mutant (as discussed, Ivanković and Đermić 2012; Đermić 2015), analogously to what was observed *in vitro* (Yu *et al.* 1998; Anderson *et al.* 1999). In this mutant, RecA loading is achieved by RecFOR proteins, which perform it from an ss-ds DNA junction (Morimatsu and Kowalczykowski 2003), *i.e.*, from the 5′-end of that tens of kb-long 3′-terminated tail (as discussed, Ivanković and Đermić 2012). Therefore, ssExos of 3′–5′ polarity likely need to digest very long ss stretches in order to reach a growing RecA nucleofilament. This is a heavy task because that ss region is overly reactive; it may get engaged in inter- and intramolecular transactions, resulting in illegitimate recombination intermediates (Ivanković and Đermić 2012) and ds secondary structures, as well as ds products of reannealing with the complementary 5′ tail, respectively. All of them may inhibit the 3′–5′ ssExos, hence increasing the longevity of the 3′-terminated tail. Furthermore, ExoI, the most potent 3′–5′ ssExo (Lovett 2011), degrades the 3′ tail with an ∼275 nt s^−1^ rate (Brody *et al.* 1986), which is around four- to eightfold slower than RecBCD helicase (and also less processive) (see below). Therefore, excessive length of the unwound 3′ strands and the mechanism of RecA loading onto them in the *recB1080* mutant seemingly protects them from degradation by ssExos, thus making RecA protection dispensable. In fact, nucleolytic degradation of 3′ overhangs by 3′–5′ ssExos is essential for survival of the *recB1080* mutant (Ivanković *et al.* 2017).

Since 3′ tail preservation is common to all the aforementioned situations, we conclude that 3′ overhangs emanating from DSBs are crucial in limiting the extent of DSB processing in *E. coli*. How do 3′ tails regulate DSB processing? Our results reveal a gradient in intensity of DNA degradation in the *recA* derivatives of wild-type, *recD*, and *recB* strains (∼95, ∼40, and ∼20% degraded DNA, respectively), which unsurprisingly correlates with the activities of their respective DSB processing helicases RecBCD, RecBC, and RecQ. The RecBCD helicase/nuclease processes at least 30 kb of DNA at ∼1000–2000 bp s^−1^ *in vitro* (Bianco *et al.* 2001; Dillingham *et al.* 2005), the RecBC helicase is about fourfold slower and less processive than RecBCD (Korangy and Julin 1993, 1994), and the RecQ helicase is even slower (∼2 bp s^−1^, Xu *et al.* 2003) and less processive than RecBC. However, this proportionality of DNA degradation with the potency of the DSB-processing helicase is disrupted in the *recB1080* mutant, whose RecB^1080^CD enzyme is equally as powerful a helicase as RecBCD (Anderson *et al.* 1999); and yet, paradoxically, DNA degradation in its *recA* derivative is very weak, not stronger than that in the parental RecA^+^ strain, and certainly weaker than those observed in the *recA recB* and *recA recD* mutants. Extensive unwound tails produced in *recB1080* cells by the powerful RecB^1080^CD helicase may certainly prevent its reloading onto dsDNA ends because RecBCD and its variants (RecBC and RecB^1080^CD) bind exclusively onto a (nearly) blunt dsDNA end (Dillingham and Kowalczykowski 2008). On the other hand, continuous DNA degradation for > 2 hr, resulting in a 20–40% degraded genome (1–2 Mbp) in *recA* derivatives of *recB* and *recD* mutants (Figure 8B) reflects perpetual processing of DSBs in those bacteria, which is possible only if their slow and poorly processive helicases (RecQ and RecBC) are repeatedly loaded onto the processed DNA ends. As a consequence, DSB processing catalyzed by repeated reloading of a weak helicase, such as RecQ or RecBC, turns out to be more vigorous than that accomplished by a single-round activity of the powerful helicase RecB^1080^CD. Consequently, we infer that RecA prevents reloading of RecQ and RecBC helicases in *recB* and *recD* mutants, respectively. Inactivation of 3′–5′ ssExos in the *recD* mutant alleviates the RecA inhibition of DNA degradation, indicating that RecA inhibits DNA degradation by protecting 3′ tails from being degraded by 3′–5′ ssExos. Preserved 3′ tails in turn inhibit RecBC reloading onto the processed DNA end. Lack of DNA degradation in the *recB* mutant indicates that an optimal substrate for the RecQ binding, a dsDNA end with a 3′ protrusion (Morimatsu and Kowalczykowski 2014), becomes inaccessible to RecQ when the tail is bound by RecA. Furthermore, our results show that 3′–5′ ssExos enable DNA degradation in wild-type cells, suggesting that RecBCD-catalyzed DSB processing is achieved through repeated reloading of the enzyme, which depends on the removal of the 3′ tails. This indicates that, in wild-type *E. coli*, 3′–5′ ssExos regularly trim RecBCD-produced post-χ 3′ tails (Figure 8A).

However, there is an alternative explanation for the stimulatory effect of 3′–5′ ssExos on DNA degradation. Namely, if RecBCD would release long, acid-precipitable oligonucleotides while processing the DNA duplex (since it has endonucleolytic rather than exonucleolytic activity), 3′–5′ ssExos may be actively trimming those oligonucleotides, thus making them acid-soluble. In this way, low acid-soluble DNA content observed in irradiated 3′–5′ ssExos-deficient cells would mask ongoing DNA degradation in them. However, this hypothesis may be ruled out since both the un- and UV-irradiated ExoI^−^ ExoVII^−^ SbcCD^−^ RecA^−^ mutants have preserved genomic DNA, as assessed by DAPI staining and genome restriction (Repar *et al.* 2013), suggesting a lack of RecBCD-catalyzed “reckless” DNA degradation.

We show here that DNA degradation in *E. coli* is inhibited by either DSB-processing helicase inactivation (*e.g.*, by *recB* mutation in otherwise wild-type cells and by RecQ inactivation in the *recB recA* mutant), or by preservation of 3′ tails, again leading to the inability of these helicases to reload on the processed dsDNA ends. Our results therefore indicate that repeated helicase loading is the main determinant of the extent of DSB processing, with 3′ overhang metabolism being the crucial factor in helicase reloading. We describe three ways by which the availability of dsDNA ends to a helicase is controlled by 3′ tails: (i) RecA polymerization onto 3′ tails either directly inhibits RecQ loading onto them in the *recB* null mutant or inhibits their degradation by the 3′–5′ ssExos, thus preventing creation of blunt dsDNA ends and, consequently, reloading of RecBC(D) onto them; (ii) the appearance of blunt dsDNA ends is prevented by inactivation of the 3′–5′ ssExos, hence making shielding RecA binding onto such a protected 3′ overhang dispensable; and (iii) in the *recB1080* mutant, lengthy DSB-derived 3′ overhangs are protected from 3′–5′ ssExos in a RecA-independent manner (instead, this is likely achieved by their involvement in transactions that produce ds regions in them), hence preventing recreation of blunt dsDNA ends. Similarly, excessively long 3′ overhangs produced during resection inhibit meiotic DSB repair in eukaryotes (Johnson *et al.* 2007).

Our collective results indicate that resection of a dsDNA end in *E. coli* proceeds until a 3′ tail of sufficient length and stability is formed, which then inhibits further end processing by preventing reloading of a DSB-processing helicase. Factors that facilitate 3′ tails’ longevity involve: (i) 3′ tails protection from degradation by 3′–5′ ssExos, by either “insulating” them with the RecA protein or by inactivation of these 3′–5′ ssExos, and (ii) their excessive length.

The 3′ tail regulation of DSB processing that we describe here is reminiscent of DNA end-resection in eukaryotes, with a short 3′ overhang produced during initial DSB resection by the Rad50 and Mre11 nuclease (orthologs of SbcCD) directing a resected end toward HR and microhomology-mediated end joining pathways and away from the nonhomologous end joining repair pathway (Truong *et al.* 2013). Similarly, the length of (and RecA binding to) the 3′ overhangs created during DNA end resection determines the equilibrium of HR and illegitimate recombination pathways in *E. coli* (Ivanković and Đermić 2012). Furthermore, SbcCD and its eukaryotic orthologs are analogously required to enable (re)loading of the main DSB processing machines [bacterial RecBCD and eukaryotic EXOI (ExoI)/BLM DNA2 (Sgs1 Dna2)] that perform long-range dsDNA end processing (as discussed recently, Đermić 2015).

Hence, one can note a common regulatory mechanism for DSB processing, with a 3′ tail produced during end resection acting as the main supervisory element by imposing a negative feedback loop on subsequent processing reactions. This mechanism ensures that processing of dsDNA ends, continues until stable and utilizable 3′ tails are produced that enable the efficient repair of DSBs in both bacteria and eukaryotes.

## References

[bib1] Anderson D. G. Kowalczykowski S. C., 1997 a The recombination hot spot, Chi, is a regulatory element that switches the polarity of DNA degradation by the RecBCD enzyme. Genes Dev. 11: 571–581. 9119222 10.1101/gad.11.5.571

[bib2] Anderson D. G. Kowalczykowski S. C., 1997 b The translocating RecBCD enzyme stimulates recombination by directing RecA protein onto ssDNA in a χ-regulated manner. Cell 90: 77–86. 9230304 10.1016/s0092-8674(00)80315-3

[bib3] Anderson D. G. Churchill J. J. Kowalczykowski S. C., 1999 A single mutation, RecB_D1080A_, eliminates RecA protein loading but not Chi recognition by RecBCD enzyme. J. Biol. Chem. 274: 27139–27144. 10480929 10.1074/jbc.274.38.27139

[bib4] Bachmann B. J., 1972 Pedigrees of some mutant strains of *Escherichia coli* K-12. Bacteriol. Rev. 36: 525–557. 4568763 10.1128/br.36.4.525-557.1972 PMC408331

[bib5] Bianco P. R. Brewer L. R. Corzett M. Balhorn R. Yeh Y., 2001 Processive translocation and DNA unwinding by individual RecBCD enzyme molecules. Nature 409: 374–378. 11201750 10.1038/35053131

[bib6] Bresler S. E. Noskin L. A. Kuzovleva N. A. Noskina I. G., 1979 Nature of the damage to *Escherichia coli* DNA induced by gamma radiation. Int. J. Radiat. Biol. 36: 289–300. 10.1080/09553007914551061 115807

[bib7] Brody R. S. Doherty K. G. Zimmerman P. D., 1986 Processivity and kinetics of the reaction of exonuclease I from Escherichia coli with polydeoxyribonucleotides. J. Biol. Chem. 261: 7136–7143. 3519606

[bib8] Capaldo F. N. Barbour S. D., 1975 DNA content, synthesis and integrity in dividing and non-dividing cells of rec- strains of *Escherichia coli* K12. J. Mol. Biol. 91: 53–66. 1102696 10.1016/0022-2836(75)90371-x

[bib9] Churchill J. J. Anderson D. G. Kowalczykowski S. C., 1999 The RecBC enzyme loads RecA protein onto ssDNA asymmetrically and independently of Chi, resulting in constitutive recombination activation. Genes Dev. 13: 901–911. 10197989 10.1101/gad.13.7.901 PMC316600

[bib10] Clark A. J., 1982 *recA* operator mutations and their usefulness. Biochimie 64: 669–675. 6291636 10.1016/s0300-9084(82)80108-9

[bib11] Dabert P. Ehrlich S. D. Gruss A., 1992 χ sequence protects against RecBCD degradation of DNA *in vivo*. Proc. Natl. Acad. Sci. USA 89: 12073–12077. 1465442 10.1073/pnas.89.24.12073 PMC50700

[bib12] Daly M. J., 2009 A new perspective on radiation resistance based on *Deinococcus radiodurans*. Nat. Rev. Microbiol. 7: 237–245. 19172147 10.1038/nrmicro2073

[bib13] Đermić D., 2006 Functions of multiple exonucleases are essential for cell viability, DNA repair and homologous recombination in *recD* mutants of *Escherichia coli*. Genetics 172: 2057–2069. 16452142 10.1534/genetics.105.052076 PMC1456380

[bib14] Đermić D., 2015 Double-strand break repair mechanisms in *Escherichia coli:* recent insights. Adv. Genomics Genet. 5: 35–42.

[bib15] Đermić D. Halupecki E. Zahradka D. Petranović M., 2005 RecBCD enzyme overproduction impairs DNA repair and homologous recombination in *Escherichia coli*. Res. Microbiol. 156: 304–311. 15808933 10.1016/j.resmic.2004.10.005

[bib16] Đermić D. Zahradka D. Petranović M., 2006 a Exonuclease requirements for recombination of λ-phage in *recD* mutants of *Escherichia coli*. Genetics 173: 2399–2402. 16702415 10.1534/genetics.106.060426 PMC1569708

[bib17] Đermić D. Đermić E. Zahradka D. Petranović M. Lerš N., 2006 b Gamma-irradiated RecD overproducers become permanent *recB^-^/recC*^-^ phenocopies for extrachromosomal DNA processing due to prolonged titration of RecBCD enzyme on damaged *Escherichia coli* chromosome. Biochimie 88: 379–386. 16377056 10.1016/j.biochi.2005.11.003

[bib18] Dillingham M. S. Kowalczykowski S. C., 2008 RecBCD enzyme and the repair of double-stranded DNA breaks. Microbiol. Mol. Biol. Rev. 72: 642–671. 19052323 10.1128/MMBR.00020-08 PMC2593567

[bib19] Dillingham M. S. Webb M. R. Kowalczykowski S. C., 2005 Bipolar DNA translocation contributes to highly processive DNA unwinding by RecBCD enzyme. J. Biol. Chem. 280: 37069–37077. 16041061 10.1074/jbc.M505520200

[bib20] Ivančić-Baće I. Peharec P. Moslavac S. Škrobot N. Salaj-Šmic E., 2003 RecFOR function is required for DNA repair and recombination in a RecA loading-deficient *recB* mutant of *Escherichia coli*. Genetics 163: 485–494. 12618388 10.1093/genetics/163.2.485 PMC1462458

[bib21] Ivanković S. Đermić D., 2012 DNA end resection controls the balance between homologous and illegitimate recombination in *Escherichia coli*. PLoS One 7: e39030. 22720024 10.1371/journal.pone.0039030 PMC3375238

[bib22] Ivanković S. Vujaklija D. Đermić D., 2017 Nucleolytic degradation of 3′-ending overhangs is essential for DNA-end resection in RecA-loading deficient *recB* mutants of *Escherichia coli*. DNA Repair (Amst.) 57: 56–65. 28689072 10.1016/j.dnarep.2017.06.024

[bib23] Jockovich M. E. Myers R. S., 2001 Nuclease activity is essential for RecBCD recombination in *Escherichia coli*. Mol. Microbiol. 41: 949–962. 11532156 10.1046/j.1365-2958.2001.02573.x

[bib24] Johnson R. Borde V. Neale M. J. Bishop-Bailey A. North M., 2007 Excess single-stranded DNA inhibits meiotic double-strand break repair. PLoS Genet. 3: e223. 18081428 10.1371/journal.pgen.0030223 PMC2098809

[bib25] Kohiyama M. Contremoulins V. Baudin X., 2013 Trashing of single-stranded DNA generated during processing of arrested replication fork in *E. coli*. J. Mol. Biol. 425: 4837–4844. 23810902 10.1016/j.jmb.2013.06.027

[bib26] Korangy F. Julin D. A., 1993 Kinetics and processivity of ATP hydrolysis and DNA unwinding by the RecBC enzyme from *Escherichia coli*. Biochemistry 32: 4873–4880. 8387820 10.1021/bi00069a024

[bib27] Korangy F. Julin D. A., 1994 Efficiency of ATP hydrolysis and DNA unwinding by the RecBC enzyme from *Escherichia coli*. Biochemistry 33: 9552–9560. 8068630 10.1021/bi00198a022

[bib28] Kuzminov A., 1999 Recombinational repair of DNA damage in *Escherichia coli* and bacteriophage λ. Microbiol. Mol. Biol. Rev. 63: 751–813. 10585965 10.1128/mmbr.63.4.751-813.1999 PMC98976

[bib29] Kuzminov A. Stahl F. W., 1997 Stability of linear DNA in *recA* mutant *Escherichia coli* cells reflects ongoing chromosomal DNA degradation. J. Bacteriol. 179: 880–889. 9006046 10.1128/jb.179.3.880-888.1997 PMC178773

[bib30] Kuzminov A. Shabtach E. Stahl F. W., 1994 χ-sites in combination with RecA protein increase survival of linear DNA in *Escherichia coli* by inactivating exoV activity of RecBCD nuclease. EMBO J. 13: 2764–2776. 8026461 10.1002/j.1460-2075.1994.tb06570.x PMC395156

[bib31] Lavery P. E. Kowalczykowski S. C., 1992 Biochemical basis of the constitutive repressor cleavage activity of recA730 protein. A comparison to recA441 and recA803 proteins. J. Biol. Chem. 267: 20648–20658. 1400384

[bib32] Little J. W., 1991 Mechanism of specific LexA cleavage-autodigestion and the role of RecA coprotease. Biochimie. 73: 411–422. 1911941 10.1016/0300-9084(91)90108-d

[bib33] Lovett S. T., 2011 The DNA exonucleases of *Escherichia coli*. EcoSal Plus 4 Available at: .http://www.asmscience.org/content/journal/ecosalplus/10.1128/ecosalplus.4.4.7 10.1128/ecosalplus.4.4.7 PMC4238392 26442508

[bib34] Miller J. H., 1992 *A Short Course in Bacterial Genetics*. Cold Spring Harbor Laboratory Press, Cold Spring Harbor, NY.

[bib35] Miranda A. Kuzminov A., 2003 Chromosomal lesion suppression and removal in *Escherichia coli* via linear DNA degradation. Genetics 163: 1255–1271. 12702673 10.1093/genetics/163.4.1255 PMC1462524

[bib36] Morimatsu K. Kowalczykowski S. C., 2003 RecFOR proteins load RecA protein onto gapped DNA to accelerate DNA strand exchange: a universal step of recombinational repair. Mol. Cell 11: 1337–1347. 12769856 10.1016/s1097-2765(03)00188-6

[bib37] Morimatsu K. Kowalczykowski S. C., 2014 RecQ helicase and RecJ nuclease provide complementary functions to resect DNA for homologous recombination. Proc. Natl. Acad. Sci. USA 111: E5133–E5142. 25411316 10.1073/pnas.1420009111 PMC4260596

[bib38] Persky N. S. Lovett S. T., 2008 Mechanisms of recombination: lessons from *E. coli*. Crit. Rev. Biochem. Mol. Biol. 43: 347–370. 19016098 10.1080/10409230802485358

[bib39] Repar J. Briški N. Buljubašić M. Zahradka K. Zahradka D., 2013 Exonuclease VII is involved in “reckless” DNA degradation in UV-irradiated *Escherichia coli*. Mutat. Res. 750: 96–104. 23123979 10.1016/j.mrgentox.2012.10.005

[bib40] Rinken R. Thoms B. Wackernagel W., 1992 Evidence that *recBC*-dependent degradation of duplex DNA in *Escherichia coli recD* mutants involves DNA unwinding. J. Bacteriol. 174: 5424–5429. 1322885 10.1128/jb.174.16.5424-5429.1992 PMC206381

[bib41] Sassanfar M. Roberts J. W., 1990 Nature of the SOS-inducing signal in *Escherichia coli*. The involvement of DNA replication. J. Mol. Biol. 212: 79–96. 2108251 10.1016/0022-2836(90)90306-7

[bib42] Schneider C. A. Rasband W. S. Eliceiri K. W., 2012 NIH Image to ImageJ: 25 years of image analysis. Nat. Methods 9: 671–675. 22930834 10.1038/nmeth.2089 PMC5554542

[bib43] Shee C. Cox B. D. Gu F. Luengas E. M. Joshi M. C., 2013 Engineered proteins detect spontaneous DNA breakage in human and bacterial cells. Elife 2: e01222. 24171103 10.7554/eLife.01222 PMC3809393

[bib44] Shinagawa H. Iwasaki H. Kato T. Nakata A., 1988 RecA protein-dependent cleavage of UmuD protein and SOS mutagenesis. Proc. Natl. Acad. Sci. USA 85: 1806–1810. 3126496 10.1073/pnas.85.6.1806 PMC279868

[bib45] Skarstad K. Boye E., 1993 Degradation of individual chromosomes in *recA* mutants of *Escherichia coli*. J. Bacteriol. 175: 5505–5509. 8366035 10.1128/jb.175.17.5505-5509.1993 PMC206606

[bib46] Symington L. S., 2014 End resection at double-strand breaks: mechanism and regulation. Cold Spring Harb. Perspect. Biol. 6: a016436. 25085909 10.1101/cshperspect.a016436 PMC4107989

[bib47] Truong L. N. Li Y. Shi L. Z., 2013 Microhomology-mediated end joining and homologous recombination share the initial end resection step to repair DNA double-strand breaks in mammalian cells. Proc. Natl. Acad. Sci. USA 110: 7720–7725. 23610439 10.1073/pnas.1213431110 PMC3651503

[bib48] Ulmer K. M. Gomez R. F. Sinskey A. J., 1979 Ionizing radiation damage to the folded chromosome of *Escherichia coli* K-12: sedimentation properties of irradiated nucleoids and chromosomal deoxyribonucleic acid. J. Bacteriol. 138: 475–485. 374388 10.1128/jb.138.2.475-485.1979 PMC218201

[bib49] Vallerga M. B. Mansilla S. F. Federico M. B. Bertolin A. P. Gottifredi V., 2015 Rad51 recombinase prevents Mre11 nuclease-dependent degradation and excessive PrimPol-mediated elongation of nascent DNA after UV irradiation. Proc. Natl. Acad. Sci. USA 112: E6624–E6633. 26627254 10.1073/pnas.1508543112 PMC4672812

[bib50] Vujaklija D. Maček B., 2012 Detecting posttranslational modifications of bacterial SSB proteins, pp. 205–218 in *Single-Stranded DNA Binding Proteins* in Methods in Molecular Biology, edited by Keck J. Humana Press, Copyright Holder Springer Science+Business Media, LLC, NY. 10.1007/978-1-62703-032-8_16 22976189

[bib51] Wallace S. S., 1998 Enzymatic processing of radiation-induced free radical damage in DNA. Radiat. Res. 150: S60–S79. 9806610

[bib52] Wigley D. B., 2013 Bacterial DNA repair: recent insights into the mechanism of RecBCD, AddAB and AdnAB. Nat. Rev. Microbiol. 11: 9–13. 23202527 10.1038/nrmicro2917

[bib53] Willets N. S. Clark A. J., 1969 Characteristics of some multiply recombination-deficient strains of *Escherichia coli*. J. Bacteriol. 100: 53–66. 10.1128/jb.100.1.231-239.1969 PMC315383 4898990

[bib54] Xu H. Q. Deprez E. Zhang A. H. Tauc P. Ladjimi M. M., 2003 The *Escherichia coli* RecQ helicase functions as a monomer. J. Biol. Chem. 278: 34925–34933. 12805371 10.1074/jbc.M303581200

[bib55] Yu M. Souaya J. Julin D. A., 1998 Identification of the nuclease active site in the multifunctional RecBCD enzyme by creation of a chimeric enzyme. J. Mol. Biol. 283: 797–808. 9790841 10.1006/jmbi.1998.2127

[bib56] Zahradka K. Buljubašić M. Petranović M. Zahradka D., 2009 Roles of ExoI and SbcCD nucleases in “reckless” DNA degradation in *recA* mutants of *Escherichia coli*. J. Bacteriol. 191: 1677–1687. 19074388 10.1128/JB.01877-07 PMC2648197

[bib57] Zelensky A. Kanaar R. Wyman C., 2014 Mediators of homologous DNA pairing. Cold Spring Harb. Perspect. Biol. 6: a016451. 25301930 10.1101/cshperspect.a016451 PMC4292155

